# Adaptation and Changes in Actin Dynamics and Cell Motility as Early Responses of Cultured Mammalian Cells to Altered Gravitational Vector

**DOI:** 10.3390/ijms23116127

**Published:** 2022-05-30

**Authors:** Zhenlin Ju, Tamlyn N. Thomas, Yi-Jen Chiu, Sakuya Yamanouchi, Yukari Yoshida, Jun-ichi Abe, Akihisa Takahashi, Jing Wang, Keigi Fujiwara, Megumi Hada

**Affiliations:** 1 Department of Bioinformatics and Computational Biology, University of Texas MD Anderson Cancer Center, Houston, TX 77030, USA; zju@mdanderson.org; 2 Department of Cardiology, University of Texas MD Anderson Cancer Center, Houston, TX 77030, USA; tamlyn_thomas@urmc.rochester.edu (T.N.T.); jabe@mdanderson.org (J.-i.A.); 3 Aab Cardiovascular Research Institute, University of Rochester Medical School, Rochester, NY 14642, USA; yijen5395@gmail.com; 4 Gunma University Heavy Ion Medical Center, Maebashi 371-8511, Japan; 3ra9di8o.y@gmail.com (S.Y.); yyukari@gunma-u.ac.jp (Y.Y.); a-takahashi@gunma-u.ac.jp (A.T.); 5 Radiation Institute for Science & Engineering, Prairie View A&M University, Prairie View, TX 77446, USA; mehada@pvamu.edu

**Keywords:** simulated microgravity, gravity sensing, RPPA, cell migration, cultured mammalian cells

## Abstract

Cultured mammalian cells have been shown to respond to microgravity (μG), but the molecular mechanism is still unknown. The study we report here is focused on molecular and cellular events that occur within a short period of time, which may be related to gravity sensing by cells. Our assumption is that the gravity-sensing mechanism is activated as soon as cells are exposed to any new gravitational environment. To study the molecular events, we exposed cells to simulated μG (SμG) for 15 min, 30 min, 1 h, 2 h, 4 h, and 8 h using a three-dimensional clinostat and made cell lysates, which were then analyzed by reverse phase protein arrays (RPPAs) using a panel of 453 different antibodies. By comparing the RPPA data from cells cultured at 1G with those of cells under SμG, we identified a total of 35 proteomic changes in the SμG samples and found that 20 of these changes took place, mostly transiently, within 30 min. In the 4 h and 8 h samples, there were only two RPPA changes, suggesting that the physiology of these cells is practically indistinguishable from that of cells cultured at 1 G. Among the proteins involved in the early proteomic changes were those that regulate cell motility and cytoskeletal organization. To see whether changes in gravitational environment indeed activate cell motility, we flipped the culture dish upside down (directional change in gravity vector) and studied cell migration and actin cytoskeletal organization. We found that compared with cells grown right-side up, upside-down cells transiently lost stress fibers and rapidly developed lamellipodia, which was supported by increased activity of Ras-related C3 botulinum toxin substrate 1 (Rac1). The upside-down cells also increased their migratory activity. It is possible that these early molecular and cellular events play roles in gravity sensing by mammalian cells. Our study also indicated that these early responses are transient, suggesting that cells appear to adapt physiologically to a new gravitational environment.

## 1. Introduction

It is now known that individual mammalian cells in culture are capable of responding to changes in gravitational forces. However, as recent reviews show, the form of responses is complex [1,2,3,4,5]. Cells respond to both hypergravity created by centrifugation [6,7,8,9] and microgravity (µG). Cellular responses to µG can be studied on the International Space Station and during short parabolic and sounding rocket flights, but these studies are not easy to perform, as there are numerous technical and cost-related issues that are difficult for ordinary investigators to overcome. For this reason, most µG studies are ground-based and performed using devices that can simulate the µG condition, such as various types of clinostats and rotating wall vessels, which are the two most commonly used devices [10,11,12]. These laboratory instruments allow experimenters to keep cells alive not only for hours, but for up to several days to propagate and differentiate them under simulated µG (SμG). Cells may then be studied by employing various molecular, biochemical, and morphological techniques.

Because available data on cultured cells’ responses to µG show a wide variability, one may ask if mammalian cells have a common gravity sensor. In plant biology, gravity sensing has a rich history, due to the fact that plants exhibit gravitropism, which has been known for over a century. As such, mechanisms for gravity response by plants and plant cells are fairly well established [13,14,15,16]. For example, the gravity-dependent sedimentation of statoliths and auxin (a plant growth hormone) is well-known. By contrast, our understanding of the cause of gravity responses by individual mammalian cells is limited, and it is not clear if each cell in our body has a gravity sensing mechanism. While many of the ground-based studies on plant cell gravibiology were conducted by changing only the direction of the gravity vector without altering its magnitude, experiments on mammalian cells were done by exposing them to SμG. What is clear at present is that cultured mammalian cells are capable of reacting to both true µG and simulated µG. This is evidenced by changes in cell shape, cytoskeletal organization, gene expression, protein modification, and many other aspects of cell physiology [2,4,17]. In addition, cellular responses depend on cell types, how the μG condition is generated, and the duration of experiments. Nevertheless, there are certain general features that are recognized in many studies, and it is possible that such common responses may be involved in gravity sensing. For example, changes in the cytoskeletal organization and activation of ion channels are often reported [2,4,17]. However, the precise molecular events for gravity sensing by individual mammalian cells are still shrouded in mystery.

The large majority of studies on μG effects on cultured cells were and are done by exposing cells to SμG for hours to days and analyzing them for changes in gene and protein expression, alterations in the genomic sequence, chromosome aberrations, and changes in cell morphology and physiology. Although these changes do qualify as cellular responses to μG, they may not be directly related to the core mechanism for cellular gravity sensing. Our study was designed to gain insights into the molecular and cell biological events that could be involved in gravity sensing by mammalian cells. We hypothesized that the gravity sensing mechanism would be activated as soon as a cell is exposed to any altered gravitational environment, not to just μG, and that the molecular and cellular changes detected in the cell would be related to the gravity sensing mechanism. Based on these assumptions, we used two completely different approaches to alter cells’ gravitational environments and tested whether we could detect similar cell responses. The first approach was to expose cultured cells to SμG for short periods of time and to analyze the cells by reverse phase protein arrays (RPPAs), which are able to identify changes in the level of protein expression and posttranslational modifications. The second approach was to flip tissue culture dishes upside down, thereby exposing cells to the reversed direction of gravity for short periods of time. This approach allowed us to observe cells under the microscope and study how the behavior of the same set of cells changes when the gravity vector is flipped without changing the magnitude of the vector. The results from these two different types of study show that cultured cells are able to respond quickly to changes in gravitational environments and that vastly different means of gravity changes induce similar responses in cells. Since many of these responses were transient, our study suggests that cells are adaptable to gravity changes and that the actin filament system that regulates cell motility plays a role in gravity sensing.

## 2. Results

### 2.1. Proteomic Characterization of Cells Exposed to Simulated Microgravity

Little is known about molecular events that take place when cultured mammalian cells are exposed to μG. Since cultured cells are able to react to changes in gravitational forces, they appear to have some mechanisms that enable them to sense such changes. It is likely that the immediate to early molecular changes that occur inside cells when the gravitational environment of cells is altered are related to gravity sensing by cells. To gain insights into cellular gravity sensing, we exposed human fibroblasts (1BR-hTERT cells) to SμG for 15 min, 30 min, 1 h, 2 h, 4 h, and 8 h and performed RPPA analyses on cell lysates made immediately after each of the SμG treatments. We chose 1BR-hTERT cells because our past studies on SμG effects on cells were done using this cell type, and the exact conditions for SμG experiments are well established [18,19]. These experiments were done in the laboratory of Dr. Akihisa Takahashi, Gunma University Heavy Ion Medical Center, Maebashi, Japan, and cell extracts were shipped on dry ice to the MD Anderson RPPA Core. In addition to the neat sample, each sample was serially diluted four times (i.e., undiluted, 1:2, 1:4, 1:8, and 1:16) and tested against 453 unique antibodies, followed by analyses by Array-Pro Analyzer 6.3 and then by SuperCurve_1.5.0 via SuperCurveGUI_2.1.1.

The heat map of the entire sample is shown in Supplemental Figure S1, which appears to exhibit several features. First, cool colors (i.e., negative to near 0 positive log2 numbers) predominate the map, indicating that there appears to be a limited number of noteworthy proteomic events detected by the antibodies we used (Supplemental Table S1). This suggests that cells did not make dramatically huge physiological changes, at least within several minutes to hours when they were exposed to SμG. Second, the “hot spots” of proteomic changes appear to stay the same throughout the duration of SμG experiments, indicating that the SμG-induced-molecular events do not hop around with time. Third, there appears to be a larger number of changes detected during the early phase of exposure to SμG. These protein modifications may be involved in gravity sensing. Fourth, the heat map shows more upregulated RPPA events than downregulated cases.

**Figure 1:**
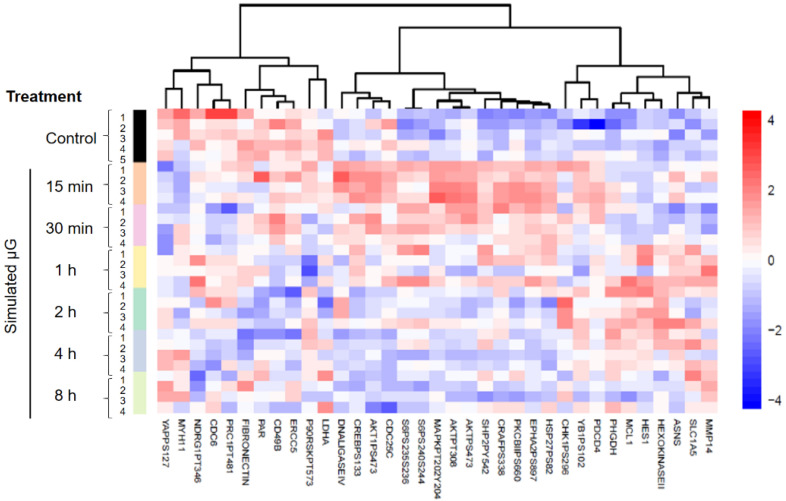
One-way supervised heatmap shows the expression patterns of the significant proteins identified by LIMMA (FDR < 0.05). Cells were treated with SμG for 15 min, 30 min, 1 h, 2 h, 4 h, and 8 h. Control samples were cells grown at 1G. Each group has over four samples.

**Figure 2:**
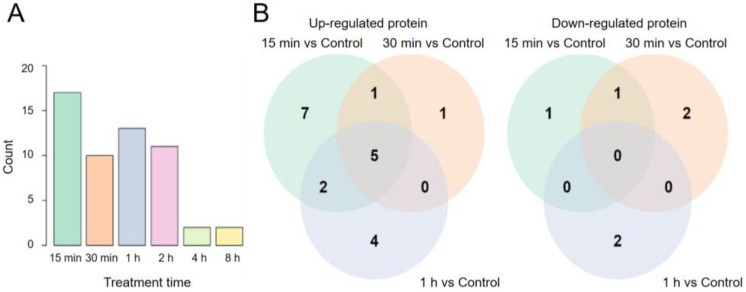
The total number of RPPA events affected by SμG in human fibroblasts (**A**) and Venn diagram showing numbers of early response proteins in cells exposed to SμG for 15 min, 30 min, and 1 h (**B**). The statistically significant proteins were identified by pair-wise comparisons between treated cells and control cells using LIMMA. FDR of < 0.05 was used as the significant level.

The baseline RPPA data from cells on the non-rotating clinostat (i.e., 1G static control cells) were compared with those from cells exposed to SμG for various lengths of time in a pair-wise comparison manner using the LIMMA method. Overall, we found that only 35 (7.7%) of 453 proteomic events were different (FDR < 0.05) in at least one group of exposed cells compared to the control, and that a burst of increased proteomic activities could be noted in the 15-min sample (Figure 1). The number of proteomic events that are statistically different between the 1G culture and the cultures exposed to SμG for 15 min up to 8 h are shown in Figure 2A. Seventeen RPPA events out of 453 independent proteomic targets examined were statistically different in cells exposed to SμG for 15 min. It is possible that 15-min exposure was not enough for cells to accumulate detectable levels of proteomic changes initiated by altered gravity. However, our analysis revealed that the number of changes in cells after 30 min of exposure to SμG were less than the number of changes after the earlier time point, with 10, 13, and 11 proteomic changes in cells exposed for 30 min, 1 h, and 2 h to SμG, respectively. This trend of reduced numbers of time-dependent proteomic changes continued, and by the exposure times of 4 h and 8 h, only two events were statistically different. The Venn diagrams (Figure 2B) show the number of proteomic events shared during the early phase of SμG exposure. There were 15 out of 17 changes that were upregulated during the first 15 min, and only two changes were downregulated during the same time period. A total of five upregulated changes lasted for 1 h. After 1 h under SμG, cells still had 11 upregulated events, seven of which were common with the 15-min cell sample. The 30-min sample shows seven upregulated and three downregulated changes. Our studies indicate that the number of proteomic changes induced by SμG is rather small and that such changes appear to be transient.

### 2.2. Proteomic Events Related to Cell Motility

Table 1 is a list of protein expression or modification (in this case phosphorylation) levels that were altered by SμG. The vertical columns show quantitative differences in proteomic events in cells exposed to SμG for different lengths of time compared with those in cells kept at 1G. Although the changes detected were small, they were statistically significant. We identified 35 changes out of 453 events tested. While most of the changes occurred early, some were detected only later. No specific proteomic change lasted for the entire period of SμG exposure. One feature that stands out is that many of the proteins with increased phosphorylation are related to cell motility and regulation of the actin filament dynamics; these proteins are identified by asterisks in Table 1: AKT [20,21], EphrinA2 [22,23], HSP27 [24,25,26], MAPK [27,28,29], PKCβII [30,31,32], S6 [33,34], SHP2 [35,36], and YB1 [37,38,39]. 

Indeed, morphological changes that are consistent with increased actin filament dynamics in cells exposed to SμG have been reported by many investigators. For example, reduced expression of stress fibers and increased lamellipodia formation were noted in various types of cultured mammalian cells maintained under SμG [40,41,42,43,44,45,46]. Thus, our RPPA results appear to provide a molecular basis for these morphological observations.

### 2.3. Live Recording of Cells Exposed to Altered Gravitational Vector: Upside-down Cell Cultures

Our RPPA study suggests that altered motility induced by the activation of actin cytoskeletal dynamics may be an early cellular response to SμG. However, it is extremely challenging to image cell dynamics, including motility, while cells are exposed to SμG, although in their recent study, Thiel et al. have successfully recorded some early morphological cellular dynamics in human macrophages under the µG condition created by a suborbital rocket flight [47]. However, it is difficult for an ordinary investigator to perform such experiments. We hypothesized that if cultured cells had a gravity sensing mechanism, this mechanism should be activated by any form of alterations in gravitational force. Mechanical force, including gravity, is a vector. In the case of SμG, the time-averaged gravity vector length (i.e., the magnitude) becomes 0, by which some mechanism in cultured cells is activated and, as the result, cells exhibit various responses. We hypothesized that this cellular mechanism should be activated if the gravity vector was altered in a different way; for example, when the directional component of the vector was altered without changing the magnitude. This can be achieved by flipping cells upside down. In this manner, cells can be studied using a microscope, and cellular responses to altered gravitational vector, especially the initial responses, can be analyzed.

Two types of cultures were used: sparse and confluent. Cells in a sparse culture should have a large degree of freedom to respond. For example, such cells will have a free cell border so that morphological events occurring at the cell border may be readily detected. In addition, they are free to move in any direction without running into another cell, at least during the early phase of locomotion. On the other hand, cells in a confluent culture have various constraints and their degree of freedom is limited. It is possible that their response may need to be coordinated with their neighbors, and such coordination may be affected by gravity. For these experiments, we used bovine aortic and human umbilical cord vein endothelial cells (BAECs and HUVECs, respectively) because we and many others have shown that endothelial cells respond to mechanical forces, such as fluid shear stress and mechanical stretch, via mechano-sensing molecules [48,49,50] and thus may respond to gravity changes in a more robust manner. Indeed, other investigators have chosen endothelial cells for the same reason to study the effects of SμG on mammalian cultured cells [9,51,52]. In addition, we used mouse 3T3 cells to represent non-endothelial cells.

### 2.4. Development of Lamellipodia

**Figure 3:**
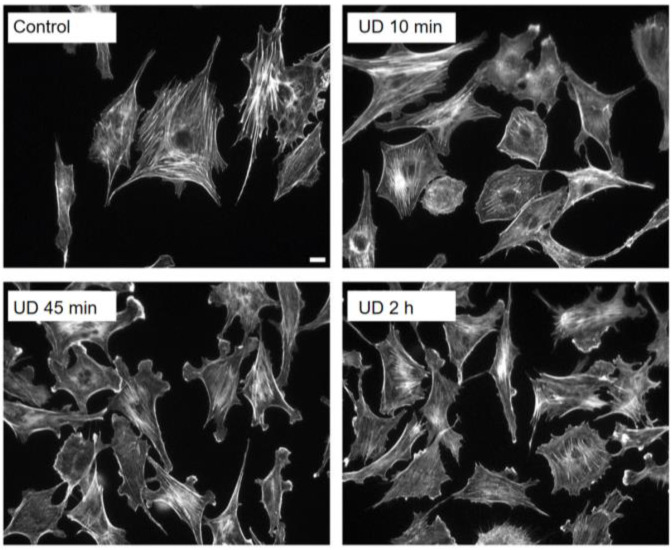
Reduced stress fibers and increased lamellipodia in upside-down cells. BAECs were stained with fluorescent phalloidin to observe actin filament organization as well as the overall cell morphology. Control cells were kept right-side up for 2 h. While control cells had many stress fibers, upside-down (UD) cells transiently lost them and extensively developed lamellipodia. These changes in upside-down cells became less noticeable by 2 h, suggesting that the morphological changes seen in upside-down cells are transient, perhaps due to the adaptation of cells to the upside-down condition. Scale bar: 10 µm.

Lamelipodia are the actively expanding cell borders and are formed at the front of migrating cells. Explosive actin polymerization occurs in lamellipodia. To study lamellipodia formation and other actin cytoskeletal reorganization in cells exposed to altered gravitational environment, we flipped sparsely cultured BAECs, HUVECs, and mouse 3T3 cells for various lengths of time, fixed them, and stained them with fluorescent phalloidin, a fluorescent marker for actin filament organization in cells. Since all these cells responded in the same manner, we illustrate the data obtained in the BAECs (Figure 3).

While cells that were not flipped upside down (Control) had well-developed stress fibers, the expression level decreased in cells that were kept upside down for 10 min. The reduced stress fiber expression caused some cells to exhibit more rounded morphology, and the cell border of many cells became less jagged (i.e., more gently curved). This trend in reduced stress fiber expression continued in upside-down cells for up to 1 h. While stress fiber expression was decreasing, there was a clear increase in the formation of lamellipodia, illustrated in cells kept upside down for 45 min (UD 45 min). Some upside-down cells developed lamellipodia within 10 min (UD 10 min), and practically all the upside-down cells exhibited lamellipodia after 30–60 min. As we noted earlier, decreased stress fiber expression and increased lamellipodia formation were observed in cells exposed to SμG [40,41,42,43,44,45,46]. Thus, it appears that the upside-down cell culture recapitulates certain microgravity responses of cells.

The morphological changes we observed in upside-down cells were transient, as cells kept upside down for longer than 1 h gradually regained stress fiber expression, began to lose lamellipodia, and showed increasingly jagged cell borders. Cells kept upside down for 2 h looked morphologically similar to the control cells (UD 2 h).

**Figure 4:**
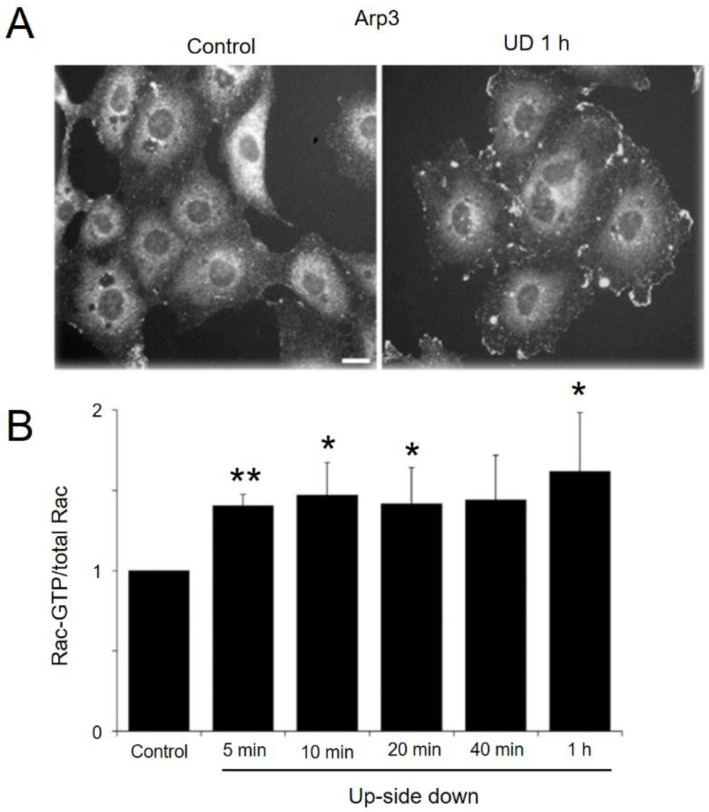
Increased lamellipodia formation in upside-down cells. (**A**) BAECs cultured right-side up (**left**) or upside down (**right**) for 1 h were stained with anti-Arp3. Note the extensive lamellipodia formation in upside-down cells. Scale bar 10 µm. (**B**) Active Rac1 (i.e., GTP bound Rac1) was quantified by the Li-Core system as described in the Methods section. Increased levels of active Rac1 in upside-down cells are shown. Mean values with standard error of the mean obtained from six independent measurements are shown. *p*-values were calculated by *t*-test between values of time 0 and values of each time point. * *p* < 0.05, ** *p* < 0.001.

To ascertain the formation of lamellipodia in upside-down cells, we stained cells kept upside down for 1 h with anti-Apr3, a marker of lamellipodia (Figure 4A). While the control cells that were not flipped showed little lamellipodia at their borders, the flipped cells had structures that stained strongly with anti-Arp3 at their cell borders. These results demonstrate a robust development of lamellipodia in upside-down cells, whose gravitational environment was altered.

Lamellipodia formation is associated with the activation of Rac1, a member of the Rho family of small GTPases [53,54]. Although Rac1 activation is localized to lamellipodia, we wondered if increased Rac1 activation could be biochemically detected in the lysate of upside-down cells, since the lamellipodia formation was so robust. As shown in Figure 4B, increased levels of Rac1 activity could be detected in upside-down cells within 5 min. Elevated Rac1 activation was observed throughout the assay period up to one hour. We detected no change in the Rac1 expression in one hour (data not shown).

### 2.5. Increased Migration of Sparsely Cultured Upside-down Cells

Lamellipodia are formed when a specific area of the cell protrudes, which usually occurs at the leading edge of migrating cells. Thus, increased lamellipodia formation is indicative of heightened cell migration. We plated cells in Sykes chambers extremely sparsely so that each cell had freedom to move about in any direction. Using a 20× lens, we chose a microscope field of view where roughly 10 cells were present, and each cell was several cell-diameter distances apart from all other cells. After recording their migration for 1h, the chamber was flipped upside down, the same field of cells was identified, and their motility was recorded for another hour. From these recordings, we generated migration paths of the same group of cells under the right-side up and upside-down conditions.

**Figure 5:**
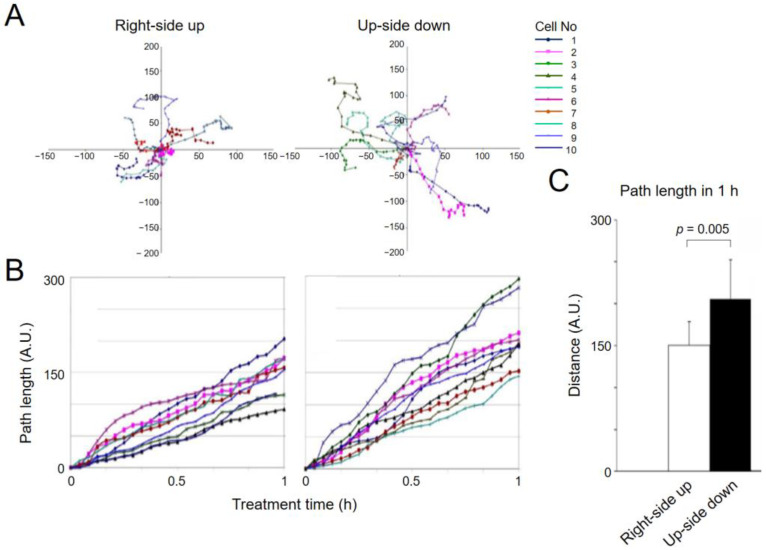
Increased migratory activity of upside-down cells. Migration of sparsely cultured BAECs in a Sykes chamber were tracked for 1 h before (**right-side up**) and after (**upside down**) flipping the chamber. The centroid positions of the nucleus of each cell at different times are plotted. (**A**) Trace of cell migration. The cell position at time 0 is superimposed at the X-Y intersection. (**B**) The data in A are replotted to show the rate of cell migration. Note that upside-down cells move faster. (**C**) Total migration path lengths of right-side up and upside-down cells. Upside-down cells exhibit increased cell migration. The migration path lengths in A.U. represent pixel numbers, and 10 A.U. corresponds approximately 1 µm. Motility assays were independently repeated three times, all showing the same effect. One set of data are presented in this figure. *p*-value was determined by *t*-test.

Figure 5A shows the migration paths of nine cells that were found in one field of view when the culture was right-side up. Each colored line represents one cell’s migration path. The original position of each cell was moved to and superimposed at the 0, 0 coordinate of the X, Y plot (Figure 5A, left). Although all cells did move, some cells exhibited the “tumbling” type of motility, staying close to the original location. The plot on the right (Figure 5A) shows cell migration traces under the reversed gravity condition. The color of lines can be used to identify the same cells under both gravitational conditions. The upside-down trace has 10 traces. This is because one cell came into the field of view while the culture was right-side up, and we included this cell in the analysis of upside-down cells. Note that the position of the cells at the end is generally farther away from the starting position, indicating that cells moved faster when they were upside down. The length of cell tracks was determined by pixel counting and indicated by using an arbitrary unit, but it can be converted into actual distance, as 10 arbitrary units (A.U.) are very close to 1 µm. The same data are plotted as the total path lengths against time (Figure 5B). The graph shows increased motility of the same set of cells when they were upside down. Figure 5C is the average distance traveled in one hour when the same group of cells were right-side up or upside down (average speeds of 15 µm/h vs 20.7 µm/h, respectively). Figure 5A also shows increased persistence of migration of upside-down cells (for example, compare the pink traces).

### 2.6. Gravity Effects on Cells in Confluent Culture

**Figure 6:**
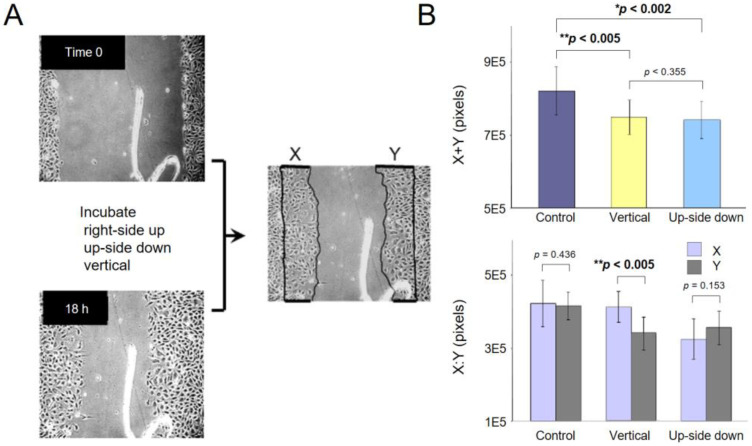
Slower wound closure in upside-down cultures. (**A**) Graphic outline of the wound healing assay. It also shows how the X and Y areas were defined. (**B**) Quantification of wound closure. *p*-values were calculated by *t*-test. These assays were repeated independently five times.

The wound healing assay was used as a migration model for cells in a confluent culture. A scratch wound was made in a confluent monolayer of BAECs in 6 cm Petri dishes, and cultures with a straight wound edge were used. On the bottom of culture dishes, we made a scratch mark so that we were able to come back to the same area of wounds for quantification. The time 0 photographs were taken from each wounded culture, including the marker, and the culture dishes were filled with fresh media, covered with Parafilm, and capped. They were then kept right-side up (control), upside down, or vertical on the dish’s side for 18 h. In vertical dishes, the wound orientation was perpendicular to the direction of gravity so that cells at the wound edge must migrate either in the direction of gravity or against it. After 18 h of culture, the same area of wound was photographed, and the re-covered area in the wound was manually determined and quantified by counting pixels within the area. Each wound had two re-covered areas, which were arbitrarily named X and Y, and the total wound repair was defined as the sum of X and Y (Figure 6A).

The total area re-covered (X + Y) in cultures kept right-side up, upside down, and vertical is quantified and graphed (Figure 6B, top). Wound healing in upside-down culture was significantly slower than the control culture. The vertical culture also showed slower healing than the control. To ascertain that both sides of the wound healed similarly, we plotted X and Y separately. Indeed, the wounds in both right-side up and upside-down cultures healed equally from both sides, albeit slower in the upside-down culture. Interestingly, however, the vertical culture showed different healing speeds of the two wounded edges. The topside of the wound healed at the same speed as the control, but the downside of the wound healed more slowly, indicating that cell migration against the direction of gravity is slower. The vertical culture is another way to show gravity effects on cells.

## 3. Discussion

Both animals and plants are able to sense and react to gravity. Various experiments have shown that gravity response occurs not only at the organismic level but also at the cellular level [1,2,3,4,5], but the mechanism for gravity sensing by individual mammalian cells is not known. To identify molecular events that may be involved in gravity sensing and the subsequent establishment of gravity responses, we employed RPPA technology on cells exposed to SµG for relatively short periods of time. We expected that some early molecular events, especially protein modifications, might be related to gravity sensing, while later molecular activities might play roles in establishing the steady state physiologic condition under µG.

Overall, it was surprising to see how small the total number of proteomic events that were different between the 1G control and the three-dimensional (3D) clinostat-treated cells was. Only a total of 35 out of 453 molecular events surveyed were found to be different in cell samples collected during the first 8 h of application of SµG. This might suggest that cells are not fundamentally reprogrammed by SµG. Our data show that although there is a noticeable number of proteomic changes at the beginning of exposure to SµG, this number gradually decreases, suggesting that cells are able to adapt to the SµG condition within several hours. Indeed, when we counted the number of molecular events in the 4 h and 8 h samples that were different from those of the control cells kept at 1G, we found only two such events, suggesting that cells under SµG for more than 4 h have the same cellular physiology as cells at 1G. Thus, our study suggests that the initial proteomic changes triggered by SµG subside within a few hours. However, many studies, including our own, have shown that SµG affects gene expression patterns [19,55,56,57] and increases chromosome aberrations [18], both of which are detected in cells kept under SµG for a period of days. It is possible that some signaling events that lead to these later events are triggered during the early phase of response, but it is also possible that some cumulative effects that develop slowly are involved. For example, gravity changes are a form of stress, which is known to activate stress responses in cells, and persistent stress may cause some of the late responses to µG. While these scenarios are based on activating or inhibiting certain specific sets of molecular events, such as certain signaling cascades, some late events may occur by chance and µG may somehow increase the probability of such events to occur. This last scenario may not be so unreasonable, because diffusion of materials is affected by µG, and this could alter the localization of soluble components of cells in a random and unpredictable manner. Reduced cytoskeletal organization by µG may also contribute to altered localization of molecules.

Table 1 shows specific proteomic changes in cells exposed to SµG. The earliest time point we have for the RPPA analysis is 15 min. Since proteins can be modified (e.g., phosphorylated) or de-modified (e.g., dephosphorylated) rapidly, the 15-min sampling point may not give sufficient temporal resolution needed to catch molecular changes that occur during gravity sensing, especially changes that happen at the beginning. However, increasing temporal resolution is technically difficult. This problem is not unique to gravity research, but is common to all mechanosensing studies. Setting up an experiment to expose cells to a mechanical stress requires the handling of cell containers, during which cells are exposed to various mechanical forces, such as mechanical shocks during the mounting of cell containers to experimental devices, changes in gravity directions, initial acceleration of forces. Thus, one must consider that very early molecular responses are a mixture of these unrelated mechanical effects plus the mechanical effect one wants to investigate. Furthermore, if mechanical stimulation is for a short time, one must also consider the contribution of forces during sample dismount. For our clinostat experiments, we felt that a sampling time shorter than 15 min would introduce too much of these unwanted mechanical stimuli. For this reason, it is possible that our study missed the critical proteomic event(s) associated with gravity sensing. Parabolic flights offer unique opportunities to study very early cell responses to gravitational changes [47,55,58].

Looking at the list of specific molecules in Table 1, there is no obvious signaling cascade in which most of the proteomic changes can be arranged in a logical sequential manner. However, many of the phosphorylated proteins in the list have been shown to promote the migration of normal cells and cancer cells [22,23,24,25,26,27,28,29,30,31,32,33,34,35,36,37,38,39]. This finding is consistent with reports by others that SµG induces rearrangements of mammalian cells’ cytoskeleton and adhesion to the extracellular matrix [2] and that SµG affects migration of cultured endothelial cells [51,52]. Studying migration of cells while they are under SµG is not an easy task. However, recent reports demonstrate that such studies are possible, although ingeniously designed new devices had to be developed for these studies [47,59]. Unfortunately, such devices are not available to many investigators. Thus, it became necessary to develop a new ground-based system with which early gravity responses of cells could be investigated. Assuming that cultured mammalian cells have a molecular mechanism that can be activated by changing one of the two vectors of gravity, this mechanism should be turned on as soon as cells are exposed to any form of gravitational changes (i.e., changes in the direction and/or the magnitude of gravitational force), not just µG changes. The system we developed is to flip cells upside down, which changes the direction of the gravity vector. If our hypothesis is correct and if we obtain cell samples soon after flipping, we should be able to capture the initial responses of cultured cells to changes in their gravitational environment. 

First, we used sparsely cultured cells in order to study cells’ full capacity to respond to gravity. In this case, stress fiber expression was downregulated, and lamellipodia formation was promoted. The same morphological changes we observed in upside-down cells have been reported by many investigators in various types of cultured cells exposed to SµG [40,41,42,43,44,45,46,57]. These results, together with ours, indicate that both SµG and the upside-down reversed gravity condition induce the same cell responses and support our hypothesis that cultured cells in general respond similarly when their gravitational environment changes. Thus, it is possible that there is a gravity-sensing mechanism in mammalian cells. The robust development of lamellipodia and the increased level of active Rac1, which was also detected in cells treated with SµG [60], are consistent with the upregulated motility of cells in a sparse culture. When the wound healing assay was used to study the motile response of confluent cells, cell motility determined by the extent of wound closure was slower in upside-down cultures. Cells in a confluent culture must first free themselves from their neighbors before they can migrate as a single cell. It is possible that changes in gravitational conditions downregulate the dissolution of cell-cell contacts or increase intercellular bonding. In vitro wound healing is also slowed down by SµG [61]. These results indicate that wound healing is affected by gravity, which may then suggest that wound healing in space may be slower.

The upside-down cultures we employed did not require expensive devices and allowed studying cells under the microscope, as well as using state-of-the-art molecular and biochemical analytical methods. We suggest that this system is particularly useful for studying the initial molecular events when the gravitational environment of the cell is altered, which then should activate the generic gravity sensing mechanism. As we discussed earlier, many of the ground-based devices used to study gravity effects on cells cannot provide data with good time resolution, especially for the early gravity response. By contrast, the upside-down system, because of its simple maneuver, is not mechanically disturbed in any significant manner (except for gravity) during experiments. Thus, it should be possible to obtain good temporal resolution of molecular events, such that it might be possible to get closer to the molecular mechanism for gravity sensing. One caveat is that the upside-down system is not a model for microgravity. Thus, the data obtained in the upside-down experiments must be validated using clinostats and other devices and means. The upside-down cell system is a very transient way to change the orientation of the gravity vector without changing the magnitude of the gravity force. We suggest that this approach may be useful in identifying molecular events that could be involved in cellular gravity sensing (i.e., the initial biochemical response of cells) and that the molecular modifications found could be studied in a targeted manner in cells under the SµG and true µG conditions.

Our findings described in this report, however, are subject to some limitations. Although our study suggests that certain molecular and cellular events that center around actin filament dynamics are induced in cultured mammalian cells by both SµG and by a reversed gravitational field, these results were obtained using several different cell types. Data from these different experiments done using a single cell type may provide more mutually relatable information on and insights into cellular gravity sensing. However, there are some positive aspects in using several different cell types. As we have indicated, other investigators’ results show reduced stress fiber expression and increased lamellipodia development in cells on clinostats [40,41,42,43,44,45,46,57]; these results come from various cell types, including cancer cells, stem cells, and primary culture cells. The fact that these different cell types respond in a similar manner is interesting, because it suggests the presence of a common mechanism in cultured cells that can be activated by SµG. Our upside-down cells, including BAECs, HUVECs, and mouse 3T3 cells, all exhibited the same morphological responses seen in SµG-treated cells by others, suggesting that this SµG-activated mechanism can be also activated by reversed gravity. Further studies are necessary to confirm this interesting idea. One urgent study that needs to be carried out is to perform RPPA analyses on upside-down cells. Such an analysis may provide further insights into the mechanism of gravity sensing.

In summary, our study has revealed two important aspects of mammalian cells’ response to gravity changes, especially with respect to early responses to sudden changes in gravitational environment. First, our RPPA data show that the proteomics of human fibroblasts exposed to SµG for 4–8 h is indistinguishable from that of cells kept at 1G. This suggests that cells are able to adapt to µG so that their physiology is no longer affected by this new gravitational environment. This was surprising to us, because we expected cells under SµG to establish a stable proteomic pattern different from that of cells at 1G. Such a new steady state proteomic pattern was not found. The second noteworthy result relates to cell motility. When cells were allowed a high degree of freedom for their response, their motile activity increased when they were exposed briefly to an altered gravitational environment. This conclusion was supported by data from the RPPA analyses. However, we found that the motility of cells in a confluent culture and exposed to a gravity change was slower when compared with the motility of 1G control cells. These results suggest that the gravity effect on cell motility must be carefully interpreted, considering the level of confluency of culture. Going forward, it is not clear how these results relate to µG effects on humans. However, our study might indicate that µG or changes in gravity vector per se may not affect the fundamental physiology of the organism, as cells seem to adapt to changes in gravity. For astronauts, the negative effects of space radiation are a much more serious health problem than µG.

## 4. Materials and Methods

### 4.1. Cells

Several lines of cultured cells were used. For RPPA studies, because the antibody library was for human antigens, we used human fibroblasts (1BR-hTERT cells). This cell line was routinely used in many of our gravity- and space-biology-related studies, in which the same 3D clinostat (described below) was used [18,19]. 1BR-hTERT cells were provided by Dr. Penny A. Jeggo (University of Sussex, Brighton, UK) and Dr. Atsushi Shibata (Gunma University Initiative for Advanced Research, Maebashi, Japan). These cells were cultured in CO_2_-independent medium (ThermoFisher Scientific, Waltham, MA, USA) supplemented with 10% (*v*/*v*) fetal bovine serum (FBS; MP Biomedicals, Santa Ana, CA, USA), 200 mM L-glutamine (ThermoFisher Scientific, Waltham, MA, USA), and penicillin–streptomycin (Nacalai Tesque, Kyoto, Japan). Cells were cultured in disposable, custom-made culture chambers (Chiyoda Co., Kanagawa, Japan) as described in Tanigawa et al. [62] for 24 h before exposing them to SμG. Human umbilical vein endothelial cells (HUVECs) were isolated by treating the lumen of umbilical cord veins with collagenase [63] and cultured in 10 cm Petri dishes coated with 0.2% gelatin type A (#901771; MP Biomedicals, Santa Ana, CA, USA) using Endothelial Cell Medium (ECM, #1001, ScienCell, Carlsbad, CA, USA) supplemented with 5% (*v*/*v*) FBS (Equitech-Bio, Kerrville, TX, USA), Endothelial Cell Growth Supplement (ECGS, #1052, ScienCell, Carlsbad, CA, USA), and penicillin-streptomycin (Mediatech, Hendon, VA, USA). HUVECs, passages between 3 and 6, were used for studies on reversed gravity (i.e., upside-down) experiments. Bovine arterial endothelial cells (BAECs) and mouse NIH3T3 cells were also used in the upside-down experiments. We used different cell types to show the biological generality of cultured mammalian cells’ response to gravity. BAECs were purchased from Clonetics (San Diego, CA, USA), cultured in Dulbecco’s modified Eagle’s medium (DMEM) (1 g/L glucose) supplemented with 10% FBS and penicillin-streptomycin, and used between passages 6 to 9. Mouse NIH3T3 cells were obtained from ATCC and were cultured in the same manner.

### 4.2. 3-D Clinostat

The custom-made 3D clinostat (Portable Microgravity Simulator PMS-CSTI; Advanced Engineering Services Co. Ltd. (AES), Ibaraki, Japan) has been described and characterized by Ikeda et al. [64] and used extensively by our group [18,19,64,65,66]. It is housed in the laboratory of Dr. Akihisa Takahashi, Gunma University Heavy Ion Medical Center, Maebashi, Gunma, Japan. Cells were grown in disposable closed cell culture chambers (DCC) described by Tanigawa et al. [62]. A suspension of cells (~10^5^ cells/mL) made in the CO_2_-independent culture medium was injected into DCC, leaving no air space or bubbles and cultured for 24 h. The clinostat can hold two chambers, and the sample stage temperature was kept to 37 °C. Semi-confluent cultures of 1BR-hTERT cells were exposed to μG for 0 min, 15 min, 30 min, 1 h, 2 h, 4 h, and 8 h. For each time point, four independent clinostat runs were performed, and samples were analyzed by RPPA separately.

### 4.3. Sample Preparation for RPPA

Immediately after cells were exposed to μG for 0–8 h, one face of the DCC chamber was cut open entirely, cells were washed twice with cold phosphate buffered saline (PBS), and the chamber was placed on ice. Ice cold lysis buffer (150 μL/chamber) containing 1% Triton X-100, 50 mM HEPES (pH 7.4), 150 mM NaCl, 1.5 mM MgCl_2_, 1 mM EGTA, 100 mM NaF, 10 mM Na pyrophosphate, 1 mM Na_3_VO_4_, 10% glycerol, and protease (Cat. #05056489001) and phosphatase (Cat. #04906837001) inhibitors from Roche Applied Science (Penzberg, Germany) was added to the chamber, and cells were extracted for 20 min with gentle shaking every 5 min. Cells were scraped off from the chamber and collected with the lysate into a microcentrifuge tube. The cell lysate was cleared by centrifugation at 16,000 × g for 10 min at 4 °C, and the supernatant was immediately frozen at −80 °C. The μG experiments were performed at Gunma University and frozen cell lysates were shipped on dry ice to the Reverse Phase Protein Array Core Facility at MD Anderson (https://www.mdanderson.org/research/research-resources/core-facilities/functional-proteomics-rppa-core.html) (accessed on 19 May 2020), where RPPA analyses were performed.

### 4.4. RPPA Experiment and Data Analysis

RPPA was performed as described previously [67,68]. Briefly, the protein concentration of cell lysates was adjusted to 1 µg/µL with 1% sodium dodecyl sulfate (SDS), and each sample lysate was serially diluted two-fold to make 5 dilutions (undiluted, 1:2, 1:4, 1:8, and 1:16) with lysis buffer. The lysates were then printed on nitrocellulose-coated slides (Grace Bio-Labs, Bend, OR, USA) using an Aushon 2470 Arrayer (Aushon BioSystems, Billerica, MA, USA). Slides were hybridized with 453 primary antibodies (Supplemental Table S1), followed by detection with secondary antibodies. Signals were visualized with a DAB colorimetric reaction and captured using a Dako Cytomation-catalyzed system (Dako, Carpinteria, CA, USA). Slides were scanned using a CanoScan 9000F (Canon U.S.A., Melville, NY, USA) and spot intensities were analyzed and quantified using ArrayPro Analyzer 6.3 (Media Cybernetics, Washington D.C., USA). SuperCurve v1.5.0 [69], which is available at (https://r-forge.r-project.org/R/?group_id=1899) (accessed on 16 June 2021), was used to estimate relative protein levels. First, the spatial bias of raw spot intensities was adjusted using “control spots” that were arrayed across the slides [70], then a fitted curve (termed as “SuperCurve”) was created with signal intensities on the Y-axis and relative log2 amounts of each protein on the X-axis using a non-parametric, monotone increasing B-spline model. Quality control (QC) of slides was performed using a logistic classifier [71] that determined a QC score for each slide, and by default only slides with QC scores above 0.8 on a 0–1 scale were used. After SuperCurve fitting, protein measurements were normalized for loading as described [68,69], using median centering across antibodies.

Statistical and bioinformatic analysis was performed using the packages of R (version 4.1.0). Bioconductor v3.13. LIMMA package [72] was used to compare protein expression between the treated and control cells. The type I error rate of multiple comparison was controlled by using false discovery rate (FDR). FDR of less than 0.05 was used as the cutoff significant level for identifying statistically significant proteins. Protein expression patterns were displayed in a hierarchical clustering heatmap, for which Spearman’s correlation was used as the correlation matrix and the Ward method was used as the linkage rule.

### 4.5. Upside-down Experiments

BAECs, mouse NIH3T3 cells, and HUVECs were sparsely plated in tissue culture Petri dishes or Sykes-Moore chambers (Bellco Glass, Vineland, NJ, USA) and cultured overnight. To perform upside-down experiments for morphological studies, we used cells cultured in Petri dishes. DMEM was used to completely fill up the dish, which was then capped with a piece of sterile Parafilm without trapping bubbles, and then the Petri dish lid was applied over the Parafilm. Cells were cultured overnight, and the sealed dishes were flipped upside down and cultured for up to 1–2 h, as indicated for each assay. For the control, sealed cultures were flipped once and returned to right-side up and cultured right-side up for up to 2 h. For cell motility assay, Sykes-Moore chambers were used as described below. For each time point, experiments were done in triplicate and repeated 3–6 times.

### 4.6. Fluorescence Microscopy

Alexa Fluor 488-labeled phalloidin (ThermoFisher Scientific, Waltham, MA, USA) was used to determine the cell shape and the actin filament organization. Cells were plated sparsely in glass bottom dishes (Nunc #150680, ThermoFisher Scientific, Waltham, MA, USA), and the upside-down treatment was performed as described above. Cells were then fixed with 3.7% formaldehyde in PBS for 10 min and permeabilized with 0.1% Triton X-100 in PBS for 1 min at room temperature (RT). Cells were stained with the fluorescently labeled phalloidin (diluted 1000 times with PBS) at RT for 15 min in a light-tight box. For Arp3 staining, cells were fixed with 1% glutaraldehyde in PHEM (60 mM Pipes, 25 mM HEPES, 10 mM EGTA, 2 mM MgCl_2_, pH 6.9) for 2 min, quenched with a saturated aqueous solution of thiocarbohydrozide for 30 min, permeabilized with 0.1% SDS in PHEM for 1 min, and blocked with 3% bovine serum albumin in TTBS (0.05% Tween, 20 mM Tris pH 7.6, 137 mM NaCl) for 30 min, all at RT. Cells were then incubated with mouse monoclonal anti-Arp3 (BD Transduction Laboratories, Franklin Lakes, NJ, USA) for 1 h at RT, followed by staining with Alexa 546-labeled goat anti-mouse IgG (ThermoFisher Scientific, Waltham, MA, USA), diluted 250 times with PBS, for 1 h at RT in a light-tight box. Control staining was done using either non-immune mouse IgG as the primary antibody or staining samples with the secondary antibodies alone. Images were observed using an Olympus BX51 epi-fluorescence microscope equipped with an RT color Spot camera (Diagnostic, Inc. Galanta, Germany), using a 60× water immersion (N.A. 0.90) lens.

### 4.7. Cell Motility Analysis

BAECs were injected into Sykes-Moore chambers with a 1 mm thick gasket and maintained in DMEM supplemented with 10% FBS and 10 mM HEPES. Time-lapse recording was carried out every 2 min using an Olympus IX81 inverted microscope with a CO_2_-humidity-temperature controlled incubation chamber (Precision Plastics, Beltsville, MD, USA), a 20× phase contrast objective lens (N.A. 0.40), and a deep-cooled CCD camera (Orca-ER, Hamamatsu Photonics, Hamamatsu, Japan). Cells were first recorded for 1 h in the right-side up orientation, flipped upside down by inverting the chamber, quickly identified, and further recorded for 1 h. Cell tracks were made by connecting the centroid position of cells and analyzed using Olympus SlideBook software. The experiments were repeated three times.

### 4.8. Rac1 Activity Assay

Semi-confluent cultures of BAECs were kept upside down for 0–60 min, and cell lysates were made in a modified Lysis/Binding/Washing buffer (25 mM Tris pH 7.5, 150 mM NaCl, 5 mM MgCl_2_, 1% NP-40, 1 mM DTT, 5% glycerol, and 1% protease inhibitor cocktail) and centrifuged at 16,000× *g* for 15 min. The supernatant was immediately incubated with 20 µg of GST-fusion protein containing the p21-binding domain (PBD) of human p21-activated protein kinase 1 (PAK1) and Swellgel Immobilized Glutathione Discs (Pierce, Rockford, IL, USA) for 1 h at 4 °C with continuous shaking. The affinity precipitated complex was washed three times with the Lysis/Binding/Washing buffer and eluted into SDS sample buffer. The eluted material was immunoblotted with mouse monoclonal anti-human Rac1 (BD Transduction Laboratories, San Jose, CA, USA). Immunoblotted bands were reacted with IRDye 800 conjugated anti-mouse IgG (Rockland, Gilbertsville, PA, USA), and signals were quantified by using the Odyssey Infrared Detection System (Li-COR Bioscience, Lincoln, NE, USA). The Rac1 activity was standardized by the total Rac1 in each cell lysate. The experiments were repeated six times.

### 4.9. Wound Healing Assay

BAECs were cultured in 6 cm Petri dishes until they formed a confluent monolayer. A single straight wound was made using a pipet tip. Cultures with a smooth straight wound edge were selected, and the bottom of each dish was marked by a pen so that the same area of the wound photographed at time 0 could be found again 18 h later. The culture dish was filled with fresh culture medium, covered with sterile Parafilm, and capped with the Petri dish lid. Wound healing was allowed to take place for 18 h, keeping the dish right-side up (control), upside down, or on its side in such a way that the wound was perpendicular to the gravity direction. Images of wounds were photographed on an Olympus IMT-2 phase contrast microscope with a 20× lens (N.A. 0.40) with an Olympus digital camera (C-5060 wide zoom). The “healed” area of the wound was quantified by the pixel counting of digital images. Experiments were repeated five times.

## Figures and Tables

**Table 1:** Fold changes (log2) of significant (FDR < 0.05) proteins in the cells exposed to SμG compared to the control cells cultured at 1G. (The extent of changes is color-coded).

Protein	Treatment Time (Minutes)					
	15	30	60	120	240	480
AKT1PS473 *	0.27					
AKTPS473 *	0.45					
AKTPT308 *	0.52	0.29				
ASNA	0.36		0.41	0.36		
CRAFPS338	0.24					
CD49B			−0.18	−0.20		
CDC35C						−0.26
CDC6		−0.16				
CHK1PS296				0.15		
CREBPS133	0.29		0.18			
DNALIGASEIV	0.25					
EPHA2PS897 *	0.99	0.83	0.70			
ERCC5				−0.16		
FIBRONECTIN				−0.54	−0.75	
HES1			0.52	0.58		
HEXOKINASEII			0.37	0.61		
HSP27PS82 *	0.44	0.37	0.36			
LDHA				−0.23		
MAPKPT202Y204 *	1.15	0.64	0.42			
MCL1				0.30		
MMP14			0.19			
MYH11	−0.23					
NDRG1PT346						−0.28
P90RSKPT573			−0.63			
PAR				−0.90		
PDCD4	0.35					
PHGDH				0.30	0.32	
PKCBIIPS660 *	0.36					
PRC1PT481		−0.15				
S6PS235S236 *	0.58	0.66	0.57			
S6PS240S244 *		0.61				
SHP2PY542 *	0.22	0.20	0.24			
SLC1A5			0.38			
YAPPS127	−0.21	−0.23				
YB1PS102 *	0.26					

* 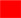 value > 1; 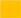, 0.5 ≦ value < 1; 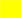, 0 ≦ value < 0.5; 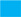, −0.5 ≦ value < 0; 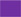, −0.5 < value.

## Data Availability

No data are publicly available due to institutional rules and regulations. Please contact corresponding authors to discuss specific data.

## Supplementary Materials

The following supporting information can be downloaded at: https://www.mdpi.com/article/10.3390/ijms23116127/s1.

## Abbreviations

1BR-hTERT cells	immortalized human fibroblasts
3D	three dimensional
A.U.	arbitrary unit
AKT	Ak strain transforming
Arp3	actin related protein 3
ATCC	American Type Culture Collection
BAEC	bovine arterial endothelial cell
CCD	charge-coupled device
DAB	3,3′-diaminobenzidine
DCC	disposable closed cell culture chamber
DMEM	Dulbecco’s modified Eagle’s medium
ECGS	endothelial cell growth supplement
ECM	endothelial cell medium
EGTA	ethylene glycol-bis(β-aminoethyl ether)-N,N,N′,N′-tetraacetic acid
FBS	fetal bovine serum
FDR	false discovery rate
GST	glutathione S-transferase
GTP	guanosine-5′-triphosphate
HEPES	4-(2-hydroxyethyl)-1-piperazineethanesulfonic acid
HSP27	heat shock protein 27
HUVEC	human umbilical vein endothelial cell
IgG	immunoglobulin G
LIMMA	linear models for microarray data
MAPK	mitogen-activated protein kinase
N.A.	numerical aperture
PAK1	p21-activated protein kinase 1
PBD	protein-binding domain
PBS	phosphate buffered saline
PKC	protein kinase C
QC	quality control
Rac1	Ras-related C3 botulinum toxin substrate 1
Rho	Ras homologous
RPPA	reverse phase protein array
RT	room temperature
S6	ribosomal protein S6
SDS	sodium dodecyl sulfate
SHP2	Src homology-2 domain-containing protein tyrosine phosphatase-2
SμG	simulated microgravity
TTBS	Tween-Tris-buffered saline
UD	upside down
μG	microgravity
YB1	Y box binding protein 1
