# Supplementary material for: Adaptation and Changes in Actin Dynamics and Cell Motility as Early Responses of Cultured Mammalian Cells to Altered Gravitational Vector
Source: Int J Mol Sci. 2022 May 30;23(11):6127. doi: 10.3390/ijms23116127 (PMC9181735; doi:10.3390/ijms23116127)
Supplement: Supplementary file 1 [file ijms-23-06127-s001.zip › ijms-1706123-supplementary.pdf]

Supplementary Materials

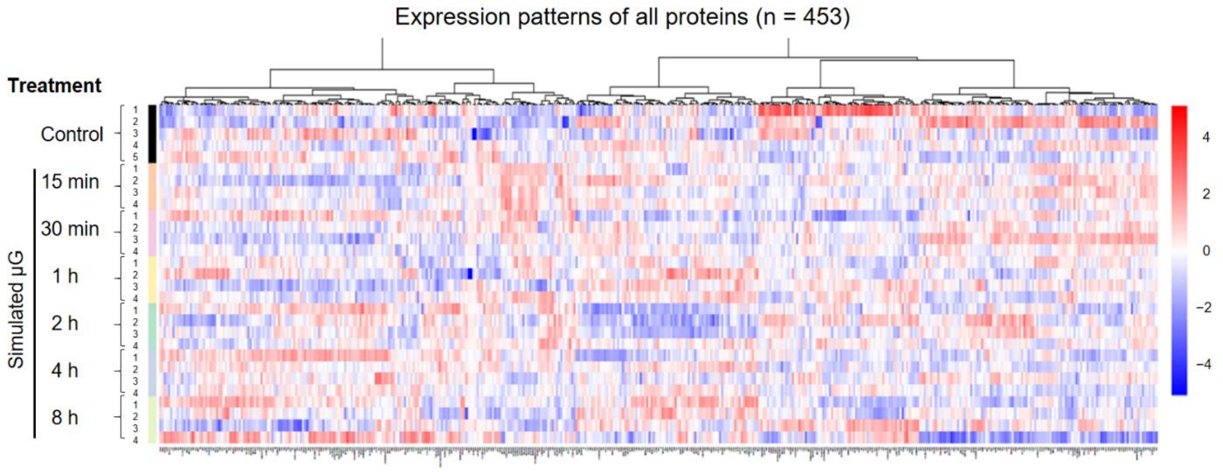

Figure S1. Heat map of all samples. Samples are identified on the right and were tested against 453 antibodies (shown at bottom and also listed in Supplemental Table S1). CO: 1G control, G15m: 15 min µG, G30m: 30 min µG, G1 h: 1h µG, G2h: 2 h µG, G4h: 4 h µG, G8h: 8 h µG.

Table S1. List of antibodies used in the RPPA study and their properties.

Slide ID	Protein Name (Antigen)	Company	Catalog Number	Gene Name	Antibody Origin	QC Score
GBL1153573	1433BETA	CST	9636	YWHAB	Rabbit	0.96545
GBL1153709	1433EPSILON	Santa Cruz	sc-23957	YWHAE	Mouse	0.89532
GBL1153648	1433ZETA	Santa Cruz	sc-1019	YWHAZ	Rabbit	0.95091
GBL1153278	4EBP1	CST	9452	EIF4EBP1	Rabbit	0.96365
GBL1153279	4EBP1PS65	CST	9456	EIF4EBP1	Rabbit	0.97243
GBL1153280	4EBP1PT37T46	CST	9459	EIF4EBP1	Rabbit	0.97445
GBL1153361	53BP1	CST	4937	TP53BP1	Rabbit	0.97609
GBL1153408	ARAF	CST	4432	ARAF	Rabbit	0.96711
GBL1153751	ARAFPS299	Invitrogen	PA5-39728	ARAF	Rabbit	0.8161
GBL1153282	ACC1	Epitomics/Abcam	1768-1/ab45174	ACACA/ACACB	Rabbit	0.96145
GBL1153281	ACCPS79	CST	3661	ACACA/ACACB	Rabbit	0.96869
GBL1153571	ACECS1	CST	3658	ACSS2	Rabbit	0.9544
GBL1153568	ACLYPS455	CST	4331	ACLY	Rabbit	0.96964
GBL1153567	ACSL1	CST	9189	ACSL1	Rabbit	0.95658
GBL1153617	ACVRL1	Invitrogen	PA5-27081	ACVRL1	Rabbit	0.81847
GBL1153682	ADAR1	Abcam	ab88574	ADAR	Mouse	0.96992
GBL1153377	AKT	CST	4691	AKT1/2/3	Rabbit	0.93288
GBL1153518	AKT1	CST	2938	AKT1	Rabbit	0.9746
GBL1153516	AKT1PS473	CST	9018	AKT1	Rabbit	0.93547
GBL1153490	AKT2	CST	3063	AKT2	Rabbit	0.9519
GBL1153517	AKT2PS474	CST	8599	AKT2	Rabbit	0.96849
GBL1153301	AKTPS473	CST	9271	AKT1/2/3	Rabbit	0.9708
GBL1153394	AKTPT308	CST	2965	AKT1/2/3	Rabbit	0.9771
GBL1153655	AMBRA1PS52	Millipore	ABC80	AMBRA1	Rabbit	0.93306
GBL1153429	AMPKA2PS345	Abcam	ab129081	PRKAA2	Rabbit	0.9445
GBL1153283	AMPKA	CST	2532	PRKAA1/2	Rabbit	0.93206
GBL1153284	AMPKAPT172	CST	2535	PRKAA1/2	Rabbit	0.97756
GBL1153685	ANNEXINI	BD Biosciences	610066	ANXA1	Mouse	0.96042
GBL1153680	ANNEXINVII	BD Biosciences	610668	ANXA7	Mouse	0.94344
GBL1153538	AR	CST	5153	AR	Rabbit	0.96786
GBL1153440	ARID1A	Sigma-Aldrich	HPA005456	ARID1A	Rabbit	0.95387

GBL1153444	ASNS	Sigma-Aldrich	HPA029318	ASNS	Rabbit	0.96921
GBL1153759	ATG3	CST	3415	ATG3	Rabbit	0.95611
GBL1153744	ATG4B	CST	13507	ATG4B	Rabbit	0.89321
GBL1153745	ATG5	CST	12994	ATG5	Rabbit	0.96002
GBL1153760	ATG7	CST	8558	ATG7	Rabbit	0.91419
GBL1153431	ATM	CST	2873	ATM	Rabbit	0.96551
GBL1153614	ATMPS1981	CST	13050	ATM	Rabbit	0.82859
GBL1153719	ATP5A	Abcam	ab14748	ATP5F1A	Mouse	0.90929
GBL1153618	ATP5H	Invitrogen	PA5-43776	ATP5PD	Rabbit	0.87088
GBL1153620	ATR	CST	13934	ATR	Rabbit	0.89572
GBL1153778	ATRX	Abcam	ab97508	ATRX	Rabbit	0.97726
GBL1153489	ATRPS428	Abcam	ab178407	ATR	Rabbit	0.96341
GBL1153555	AURORAA	CST	14475	AURKA	Rabbit	0.98021
GBL1153791	AURO-<br>RAABCPT288PT232PT198	CST	2914	AURKA-C	Rabbit	0.93851
GBL1153641	AURORAB	Invitrogen	MA5-27890	AURKB	Rabbit	0.90041
GBL1153417	AXL	CST	8661	AXL	Rabbit	0.95849
GBL1153616	BACTIN	CST	8457	ACTB	Rabbit	0.87837
GBL1153288	BETACATENIN	CST	9562	CTNNB1	Rabbit	0.97135
GBL1153401	BCATENINPT41S45	CST	9565	CTNNB1	Rabbit	0.95649
GBL1153524	BRAF	CST	14814	BRAF	Rabbit	0.92809
GBL1153291	BRAFPS445	CST	2696	BRAF	Rabbit	0.97484
GBL1153738	B7H3	CST	14058	CD276	Rabbit	0.91847
GBL1153739	B7H4	CST	14572	VTCN1	Rabbit	0.94292
GBL1153285	BADPS112	CST	9291	BAD	Rabbit	0.97555
GBL1153286	BAK	Epitomics/Abcam	1542-1/ab32371	BAK1	Rabbit	0.96772
GBL1153715	BAP1	Santa Cruz	sc-28383	BAP1	Mouse	0.93824
GBL1153287	BAX	CST	2772	BAX	Rabbit	0.9769
GBL1153289	BCLXL	CST	2762	BCL2L1	Rabbit	0.96329
GBL1153771	BCL2	CST	3498	BCL2	Rabbit	0.87461
GBL1153420	BCL2A1	Abnova	PAB8528	BCL2A1	Rabbit	0.92634
GBL1153792	BECLIN	ThermoFisher	PA1-16857	BECN1	Rabbit	0.89297
GBL1153290	BID	CST	2002	BID	Rabbit	0.96639
GBL1153402	BIM	CST	2933	BCL2L11	Rabbit	0.95022
GBL1153691	BIPGRP78	BD Biosciences	610978	HSPA5	Mouse	0.92934
GBL1153658	BMK1ERK5PT218Y220	Millipore	07-507	MAPK7	Rabbit	0.95646
GBL1153707	BRCA1	Millipore	OP92	BRCA1	Mouse	0.95687
GBL1153776	BRD4	CST	13440	BRD4	Rabbit	0.89249
GBL1153775	CABL	CST	2862	ABL1	Rabbit	0.94562
GBL1153392	CABLPY412	CST	2865	ABL1	Rabbit	0.95843
GBL1153761	CIAP	CST	3130	BIRC3	Rabbit	0.94486
GBL1153596	CJUNPS73	CST	3270	JUN	Rabbit	0.94804
GBL1153295	CKIT	Abcam	ab216450	KIT	Rabbit	0.9267
GBL1153338	CMETPY1234Y1235	CST	3129	MET	Rabbit	0.94772
GBL1153650	CMYC	Santa Cruz	sc-764	MYC	Rabbit	0.92547
GBL1153406	CRAF	Millipore	04-739	RAF1	Rabbit	0.96031
GBL1153296	CRAFPS338	CST	9427	RAF1	Rabbit	0.96504
GBL1153782	CA9	CST	5649	CA9	Rabbit	0.86359
GBL1153757	CALNEXIN	CST	2679	CANX	Rabbit	0.88957
GBL1153362	CASPASE3CLEAVED	CST	9661	CASP3	Rabbit	0.88797
GBL1153292	CASPASE7CLEAVEDD198	CST	9491	CASP7	Rabbit	0.96442
GBL1153677	CASPASE8	CST	9746	CASP8	Mouse	0.95105
GBL1153293	CAVEOLIN1	CST	3238	CAV1	Rabbit	0.95165
GBL1153527	CD134	CST	61637	TNFRSF4	Rabbit	0.95267
GBL1153728	CD171	Biolegend	826701	L1CAM	Mouse	0.94503
GBL1153662	CD20	Invitrogen	PA5-16701	MS4A1	Rabbit	0.92303

GBL1153421	CD26	Abcam	ab28340	DPP4	Rabbit	0.87677
GBL1153684	CD29	BD Biosciences	610467	ITGB1	Mouse	0.88202
GBL1153703	CD31	Dako/Fisher	M0823/MS353S0	PECAM1	Mouse	0.93074
GBL1153536	CD38	Abcam	ab108403	CD38	Rabbit	0.92537
GBL1153528	CD4	Abcam	ab133616	CD4	Rabbit	0.9395
GBL1153574	CD44	CST	37259	CD44	Rabbit	0.96672
GBL1153723	CD45	Dako	M070101-2	PTPRC	Mouse	0.85909
GBL1153676	CD49B	BD Biosciences	611016	ITGA2	Mouse	0.96091
GBL1153731	CD5	Santa Cruz	sc-393087	CD5	Mouse	0.93077
GBL1153698	CD68	R&D Systems	MAB2040	CD68	Mouse	0.95357
GBL1153665	CD74	Santa Cruz	sc-6262	CD74	Mouse	0.92217
GBL1153502	CDC25C	CST	4688	CDC25C	Rabbit	0.95243
GBL1153488	CDC2PY15	CST	4539	CDK1	Rabbit	0.9648
GBL1153424	CDC42	CST	4651	CDC42/RAC1	Rabbit	0.95796
GBL1153582	CDC6	CST	3387	CDC6	Rabbit	0.95255
GBL1153471	CDK1PT14	Abcam	ab32384	CDK1/2/3	Rabbit	0.95337
GBL1153580	CDKN2A	CST	92803	CDKN2A	Rabbit	0.93103
GBL1153753	CDT1	CST	8064	CDT1	Rabbit	0.94066
GBL1153789	CHD1L	CST	13460	CHD1L	Rabbit	0.95774
GBL1153683	CHK1	CST	2360	CHEK1	Mouse	0.9707
GBL1153428	CHK1PS296	Abcam	ab79758	CHEK1	Rabbit	0.97213
GBL1153355	CHK1PS345	CST	2348	CHEK1	Rabbit	0.96815
GBL1153663	CHK2	CST	3440	CHEK2	Mouse	0.96023
GBL1153294	CHK2PT68	CST	2197	CHEK2	Rabbit	0.94677
GBL1153520	CIITA	CST	3793	CIITA	Rabbit	0.94552
GBL1153661	CLAUDIN7	Millipore	ABT47	CLDN7	Rabbit	0.92666
GBL1153470	COG3	ProteinTech	11130-1-AP	COG3	Rabbit	0.97389
GBL1153645	COLLAGENVI	Santa Cruz	sc-20649	COL6A1	Rabbit	0.96511
GBL1153712	COMPLEXIISUBUNIT	Life Technologies	459230	SDHB	Mouse	0.94944
GBL1153777	CONNEXIN43	CST	3512	GJA1	Rabbit	0.8521
GBL1153779	COUPTFII	CST	6434	NR2F2	Rabbit	0.97249
GBL1153380	COXIV	CST	4850	COX4I1	Rabbit	0.86133
GBL1153409	COX2	CST	12282	PTGS2	Rabbit	0.94217
GBL1153750	CREB	CST	9197	CREB1	Rabbit	0.92193
GBL1153557	CREBPS133	CST	9198	CREB1	Rabbit	0.97412
GBL1153532	CSK	CST	4980	CSK	Rabbit	0.9667
GBL1153534	CTIP	CST	9201	RBBP8	Rabbit	0.96002
GBL1153298	CYCLINB1	Epitomics/Abcam	1495-1/ab32053	CCNB1	Rabbit	0.96866
GBL1153549	CYCLIND1	Millipore Sigma	SAB4502603	CCND1	Rabbit	0.95862
GBL1153664	CYCLIND3	CST	2936	CCND3	Mouse	0.9668
GBL1153752	CYCLINE1	CST	20808	CCNE1	Rabbit	0.89883
GBL1153717	CYCLOPHILINF	Abcam	ab110324	PPIF	Mouse	0.91772
GBL1153434	DATUBULIN	Abcam	ab48389	TUBA4A/TUBA3C	Rabbit	0.94059
GBL1153695	DAPK1PS308	GeneTex	GTX10524	DAPK1	Mouse	0.9737
GBL1153740	DAPK2	Abcam	ab51601	DAPK2	Rabbit	0.87249
GBL1153513	DDB1	CST	6998	DDB1	Rabbit	0.96798
GBL1153587	DDR1	CST	5583	DDR1	Rabbit	0.94892
GBL1153786	DDR1PY513	Millipore Sigma	SAB4504671	DDR1	Rabbit	0.90777
GBL1153353	DJ1	Abcam	ab76008	PARK7	Rabbit	0.96743
GBL1153700	DMK9HISTONEH3	Abcam	ab1220	HC31-4/6-8/10-12	Mouse	0.94093
GBL1153521	DNALIGASEIV	CST	14649	LIG4	Rabbit	0.9533
GBL1153570	DNAPOLG	CST	13609	POLG	Rabbit	0.973
GBL1153563	DNMT1	CST	5032	DNMT1	Rabbit	0.96667
GBL1153572	DRP1	CST	5391	DNM1L	Rabbit	0.96634
GBL1153436	DUSP4	CST	5149	DUSP4	Rabbit	0.9739

GBL1153504	DUSP6	Abcam	ab76310	DUSP6	Rabbit	0.95975
GBL1153358	DVL3	CST	3218	DVL3	Rabbit	0.95695
GBL1153772	ECADHERIN	GeneTex	GTX33614	CDH1	Rabbit	0.94354
GBL1153627	E2F1	CST	3742	E2F1	Rabbit	0.82782
GBL1153370	EEF2	CST	2332	EEF2	Rabbit	0.97682
GBL1153371	EEF2K	CST	3692	EEF2K	Rabbit	0.97538
GBL1153382	EGFR	CST	2232	EGFR	Rabbit	0.94554
GBL1153299	EGFRPY1173	Epitomics/Abcam	1124-1/ab32578	EGFR	Rabbit	0.97363
GBL1153337	EIF4E	CST	9742	EIF4E	Rabbit	0.9725
GBL1153501	EIF4EPS209	Abcam	ab76256	EIF4E	Rabbit	0.86337
GBL1153383	EIF4G	CST	2498	EIF4G1	Rabbit	0.95939
GBL1153300	ELK1PS383	CST	9181	ELK1	Rabbit	0.9674
GBL1153722	EMA	DAKO	M061329-2	MUC1	Mouse	0.95005
GBL1153558	ENOLASE1	CST	3810	ENO1	Rabbit	0.97322
GBL1153559	ENOLASE2	CST	8171	ENO2	Rabbit	0.97236
GBL1153686	ENY2	GeneTex	GTX629542	ENY2	Mouse	0.96465
GBL1153599	EPHA2	CST	6997	EPHA2	Rabbit	0.95024
GBL1153600	EPHA2PS897	CST	6347	EPHA2	Rabbit	0.9568
GBL1153601	EPHA2PY588	CST	12677	EPHA2	Rabbit	0.95384
GBL1153505	ERALPHA	CST	13258	ESR1	Rabbit	0.96943
GBL1153302	ERALPHAPS118	Epitomics/Abcam	1091-1/ab32396	ESR1	Rabbit	0.92178
GBL1153725	ERCC1	CST	5437	ERCC1	Mouse	0.94376
GBL1153430	ERCC5	Proteintech Group	11331-1-AP	ERCC5	Rabbit	0.97707
GBL1153539	ERK5	CST	3552	MAPK7	Rabbit	0.9497
GBL1153569	ERRALPHA	CST	13826	ESRRA	Rabbit	0.96424
GBL1153651	ETS1	Bethyl	A303-501A	ETS1	Rabbit	0.94131
GBL1153636	EVI1	CST	2593	MECOM	Rabbit	0.95927
GBL1153632	FABP5	Proteintech	12348-1-AP	FABP5	Rabbit	0.89735
GBL1153303	FAK	CST	3285	PTK2	Rabbit	0.95453
GBL1153621	FAKPY397	CST	8556	PTK2	Rabbit	0.91434
GBL1153395	FASN	CST	3180	FASN	Rabbit	0.97025
GBL1153525	FGFBASIC	VWR/Preprotech	10775-082/500-P18	FGF2	Rabbit	0.9532
GBL1153304	FIBRONECTIN	Epitomics/Abcam	1574-1/ab45688	FN1	Rabbit	0.92775
GBL1153509	FN14	CST	4403	TNFRSF12A	Rabbit	0.96923
GBL1153622	FOXM1	CST	20459	FOXM1	Rabbit	0.88009
GBL1153546	FOXO3	CST	12829	FOXO3	Rabbit	0.97635
GBL1153642	FOXO3APS318S321	Biorbyt	orb6051	FOXO3	Rabbit	0.91601
GBL1153630	FRS2ALPHAPY196	Invitrogen	PA5-64616	FRS2	Rabbit	0.92992
GBL1153613	G6PD	CST	12263	G6PD	Rabbit	0.92053
GBL1153359	GAB2	CST	3239	GAB2	Rabbit	0.97377
GBL1153268	GAPDH	Invitrogen	AM4300	GAPDH	Mouse	0.89347
GBL1153706	GATA3	BD Biosciences	558686	GATA3	Mouse	0.92507
GBL1153550	GATA6	CST	5851	GATA6	Rabbit	0.90725
GBL1153768	GCLC	Proteintech Group	12601-1-AP	GCLC	Rabbit	0.95681
GBL1153741	GCLM	Invitrogen	MA5-32783	GCLM	Rabbit	0.90323
GBL1153415	GCN5L2	CST	3305	KAT2A	Rabbit	0.91495
GBL1153733	GGPS1	Santa Cruz	sc-271680	GGPS1	Mouse	0.93174
GBL1153270	GLI1	CST	3538	GLI1	Rabbit	0.92575
GBL1153443	GLI3	Abcam	ab181130	GLI3	Rabbit	0.9259
GBL1153552	GLUTAMATED12	Novus	NBP2-16679	GLUD1	Rabbit	0.96192
GBL1153442	GLUTAMINASE	Abcam	ab156876	GLS	Rabbit	0.95399
GBL1153493	GRANZYMEB	CST	4275	GZMB	Rabbit	0.90488
GBL1153535	GRB7	Abcam	ab183737	GRB7	Rabbit	0.95058
GBL1153704	GSK3AB	Santa Cruz	sc-7291	GSK3A/GSK3B	Mouse	0.9289
GBL1153305	GSK3ALPHABETAPS21S9	CST	9331	GSK3A/GSK3B	Rabbit	0.96938
GBL1153398	GSK3B	CST	9315	GSK3B	Rabbit	0.96803

GBL1153367	GYS	CST	3886	GYS1	Rabbit	0.95711
GBL1153368	GYSPS641	CST	3891	GYS1	Rabbit	0.95557
GBL1153427	H2AXPS139	CST	9718	H2AX	Rabbit	0.94022
GBL1153699	H2AXPS140	Abcam	ab22551	H2AX	Mouse	0.96396
GBL1153711	HER2	Lab Vision	MS-325-P1	ERBB2	Mouse	0.82006
GBL1153373	HER2PY1248	R&D systems	AF1768	ERBB2	Rabbit	0.89224
GBL1153649	HER3	Santa Cruz	sc-285	ERBB3	Rabbit	0.94564
GBL1153339	HER3PY1298	CST	4791	ERBB3	Rabbit	0.96399
GBL1153352	HEREGULIN	CST	2573	NRG1	Rabbit	0.95487
GBL1153271	HES1	CST	11988	HES1	Rabbit	0.97419
GBL1153561	HEXOKINASEI	CST	2024	HK1	Rabbit	0.97634
GBL1153607	HEXOKINASEII	Abcam	ab228819	HK2	Rabbit	0.92167
GBL1153579	HIF1ALPHA	CST	36169	HIF1A	Rabbit	0.95558
GBL1153414	HISTONEH3	Abcam	ab1791	H3C1-4/6-8/10-12	Rabbit	0.88024
GBL1153562	HISTONEH3PS10	CST	3377	H3C1-4/6-8/10-12	Rabbit	0.96993
GBL1153644	HLADQA1	Abcam	ab211930	HLA-DQA1	Rabbit	0.93842
GBL1153277	HLADRDPDQDX	Invitrogen	PA5-29814	HLA-DRA	Rabbit	0.95666
GBL1153269	HMHA1	ProteinTech	14832-1-AP	ARHGAP45	Rabbit	0.93457
GBL1153629	HNRNPK	CST	9081	HNRNPK	Rabbit	0.9463
GBL1153666	HSP27	CST	2402	HSBP1	Mouse	0.95476
GBL1153306	HSP27PS82	CST	2401	HSBP1	Rabbit	0.95607
GBL1153494	HSP60	CST	12165	HSPD1	Rabbit	0.93175
GBL1153307	HSP70	CST	4872	HSPA1A	Rabbit	0.95478
GBL1153533	IDO	CST	86630	IDO1	Rabbit	0.93055
GBL1153410	IGF1RPY1135Y1136	CST	3024	IGF1R/INSR	Rabbit	0.93667
GBL1153308	IGFBP2	CST	3922	IGFBP2	Rabbit	0.96304
GBL1153674	IGFBP3	BD Biosciences	611504	IGFBP3	Mouse	0.94263
GBL1153309	IGFRB	CST	3027	IGF1R	Rabbit	0.97398
GBL1153506	IL6	CST	12153	IL6	Rabbit	0.96398
GBL1153623	INPP4B	CST	14543	INPP4B	Rabbit	0.96901
GBL1153273	IRB	CST	3025	INSR	Rabbit	0.97461
GBL1153652	IRF1	CST	8478	IRF1	Rabbit	0.88088
GBL1153643	IRF3	CST	4302	IRF3	Rabbit	0.95819
GBL1153345	IRS1	Millipore	06-248	IRS1	Rabbit	0.97031
GBL1153310	IRS2	CST	4502	IRS2	Rabbit	0.97417
GBL1153727	JAB1	Santa Cruz	sc-13157	COPS5	Mouse	0.93089
GBL1153437	JAGGED1	Abcam	ab109536	JAG1	Rabbit	0.96187
GBL1153400	JAK2	CST	3230	JAK2	Rabbit	0.97076
GBL1153609	JNK2	Invitrogen	PA5-28262	MAPK9	Rabbit	0.9384
GBL1153311	JNKPT183Y185	CST	4671	MAPK8	Rabbit	0.95681
GBL1153541	LAD1	Atlas	HPA028732	LAD1	Rabbit	0.96265
GBL1153656	LASU1	Bethyl	IHC-00439	HUWE1	Rabbit	0.94443
GBL1153762	LC3AB	CST	4108	MAP1LC3A/B	Rabbit	0.94451
GBL1153312	LCK	CST	2752	LCK	Rabbit	0.9656
GBL1153360	LDHA	CST	3582	LDHA	Rabbit	0.94809
GBL1153432	LRP6PS1490	CST	2568	LRP6	Rabbit	0.95558
GBL1153602	LYN	CST	2796	LYN	Rabbit	0.92456
GBL1153624	MAPKPT202Y204	CST	4370	MAPK1/MAPK3	Rabbit	0.91723
GBL1153411	MCL1	CST	5453	MCL1	Rabbit	0.88991
GBL1153734	MCT4	Millipore	AB3314P	SLC16A3	Rabbit	0.92754
GBL1153399	MDM2PS166	CST	3521	MDM2	Rabbit	0.96803
GBL1153313	MEK1	Epitomics/Abcam	1235-1/ab32576	MAP2K1	Rabbit	0.96793
GBL1153780	MEK1PS217S221	CST	9154	MAP2K1/MAP2K2	Rabbit	0.88715
GBL1153413	MEK2	CST	9125	MAP2K2	Rabbit	0.93932
GBL1153529	MELANA	Abcam	ab51061	MLANA	Rabbit	0.88895

GBL1153530	MELANOMAGP100	Abcam	ab137078	PMEL	Rabbit	0.8828
GBL1153507	MERIT40	CST	12711	BABAM1	Rabbit	0.96149
GBL1153508	MERIT40PS29	CST	12110	BABAM1	Rabbit	0.96865
GBL1153369	MERLIN	Novus	22710002	NF2	Rabbit	0.96619
GBL1153653	MIF	Santa Cruz	sc-20121	MIF	Rabbit	0.92097
GBL1153678	MIG6	CST	2440	ERRFI1	Mouse	0.96505
GBL1153566	MITF	CST	12590	MITF	Rabbit	0.9355
GBL1153495	MITOFUSIN1	CST	14739	MFN1	Rabbit	0.95515
GBL1153496	MITOFUSIN2	CST	11925	MFN2	Rabbit	0.9703
GBL1153701	MLH1	CST	3515	MLH1	Mouse	0.95896
GBL1153749	MLKL	CST	14993	MLKL	Rabbit	0.95053
GBL1153565	MMP14	Abcam	ab51074	MMP14	Rabbit	0.96717
GBL1153314	MMP2	CST	87809	MMP2	Rabbit	0.95424
GBL1153363	MNK1	CST	2195	MKNK1	Rabbit	0.97464
GBL1153729	MR1	Santa Cruz	sc-377312	MR1	Mouse	0.89888
GBL1153274	MRAP	Abcam	ab103319	MRAP	Rabbit	0.88386
GBL1153554	MSH2	CST	2017	MSH2	Rabbit	0.972
GBL1153372	MSH6	Novus	22030002	MSH6	Rabbit	0.96916
GBL1153476	MSI2	Abcam	ab76148	MSI2	Rabbit	0.97476
GBL1153721	MTCO1	Abcam	ab14705	MT-CO1	Mouse	0.90887
GBL1153315	MTOR	CST	2983	MTOR	Rabbit	0.96703
GBL1153316	MTORPS2448	CST	2971	MTOR	Rabbit	0.97663
GBL1153688	MTSS1	Novus	H00009788-M01A	MTSS1	Mouse	0.94026
GBL1153564	MYH11	GeneTex	GTX131414	MYH11	Rabbit	0.93748
GBL1153469	MYOSINIIA	CST	3403	MYH9	Rabbit	0.84636
GBL1153397	MYOSINIIAPS1943	CST	5026	MYH9	Rabbit	0.93166
GBL1153492	MYT1	CST	4282	PKMYT1	Rabbit	0.95563
GBL1153578	NCADHERIN	CST	13116	CDH2	Rabbit	0.95905
GBL1153714	NRAS	Santa Cruz	sc-31	NRAS	Mouse	0.93659
GBL1153418	NAPSINA	Epitomics/Abcam	5795-1/ab129189	NAPSA	Rabbit	0.86039
GBL1153385	NDRG1PT346	CST	3217	NDRG1	Rabbit	0.95001
GBL1153608	NDUFB4	Invitrogen	PA5-45913	NDUFB4	Rabbit	0.8648
GBL1153317	NFKBP65PS536	CST	3033	RELA	Rabbit	0.97404
GBL1153625	NOTCH1	CST	3608	NOTCH1	Rabbit	0.96808
GBL1153276	NOTCH1CLEAVED	CST	4147	NOTCH1	Rabbit	0.95321
GBL1153646	NOTCH3	Santa Cruz	sc-5593	NOTCH3	Rabbit	0.95287
GBL1153694	NQO1	CST	3187	NQO1	Mouse	0.90534
GBL1153438	NRF2	CST	12721	NFE2L2	Rabbit	0.97432
GBL1153474	4-Oct	CST	2750	POU5F1	Rabbit	0.96504
GBL1153323	PCADHERIN	CST	2130	CDH3	Rabbit	0.97215
GBL1153551	P21	Invitrogen	MA5-14949	CDKN1A	Rabbit	0.91191
GBL1153354	P27KIP1	Abcam	ab206927	CDKN1B	Rabbit	0.95185
GBL1153349	P27PT157	R&D Systems	AF1555	CDKN1B	Rabbit	0.93441
GBL1153351	P27PT198	Abcam	ab64949	CDKN1B	Rabbit	0.96765
GBL1153681	P38A	CST	9228	MAPK14	Mouse	0.96359
GBL1153318	P38MAPK	CST	9212	MAPK11/12/14	Rabbit	0.97999
GBL1153350	P38PT180Y182	CST	9215	MAPK11/12/13/14	Rabbit	0.9197
GBL1153381	P4442MAPK	CST	4695	MAPK1/MAPK3	Rabbit	0.95448
GBL1153319	P53	CST	9282	TP53	Rabbit	0.95828
GBL1153320	P70S6K1	Epitomics/Abcam	1494-1/ab32529	RPS6KB1	Rabbit	0.97097
GBL1153321	P70S6KPT389	CST	9205	RPS6KB1	Rabbit	0.97169
GBL1153615	P90RSKPT573	Invitrogen	PA5-37728	RPS6KA1	Rabbit	0.96286
GBL1153667	PAI1	BD Biosciences	612024	SERPINE1	Mouse	0.95291
GBL1153423	PAICS	Sigma-Aldrich	HPA035895	PAICS	Rabbit	0.97429
GBL1153497	PAK1	CST	2602	PAK1	Rabbit	0.92467
GBL1153435	PAK4	Invitrogen	PA5-69540	PAK4	Rabbit	0.8819

GBL1153433	PAR	Trevigen	4336-BPC-100	[PAR Modification]	Rabbit	0.95334
GBL1153540	PARG	CST	66564	PARG	Rabbit	0.95979
GBL1153537	PARP1	CST	9532	PARP1	Rabbit	0.96448
GBL1153275	PATCHED	Abcam	ab53715	PTCH1	Rabbit	0.91324
GBL1153633	PAX6	CST	60433	PAX6	Rabbit	0.902
GBL1153606	PAX8	Novus Biologicals	NBP1-32440	PAX8	Rabbit	0.95709
GBL1153322	PAXILLIN	CST	2542	PXN	Rabbit	0.96725
GBL1153693	PCNA	CST	2586	PCNA	Mouse	0.95941
GBL1153612	PD1	GeneTex	GTX128436	PDCD1	Rabbit	0.89392
GBL1153347	PDCD4	Rockland	600-401-965	PDCD4	Rabbit	0.96751
GBL1153690	PDH	Abcam	ab110332	DLAT	Mouse	0.95016
GBL1153498	PDHA1	CST	3205	PDHA1	Rabbit	0.89429
GBL1153763	PDHK1	CST	3820	PDK1	Rabbit	0.9585
GBL1153324	PDK1	CST	3062	PDPK1	Rabbit	0.94349
GBL1153325	PDK1PS241	CST	3061	PDPK1	Rabbit	0.97487
GBL1153364	PEA15	CST	2780	PEA15	Rabbit	0.9733
GBL1153365	PEA15PS116	Invitrogen	PA5-38314	PEA15	Rabbit	0.9476
GBL1153604	PERK	CST	3192	EIF2AK3	Rabbit	0.97577
GBL1153764	PHGDH	CST	13428	PHGDH	Rabbit	0.9463
GBL1153585	PHLPP	Proteintech	22789-1-AP	PHLPP1	Rabbit	0.96247
GBL1153346	PI3KP110A	CST	4255	PIK3CA	Rabbit	0.95775
GBL1153720	PI3KP110B	Santa Cruz	sc-376412	PIK3CB	Mouse	0.91841
GBL1153660	PI3KP85	Millipore	ABS1856	PIK3R1	Rabbit	0.94695
GBL1153473	PKAA	CST	5675	PRKAR1A	Rabbit	0.96261
GBL1153439	PKCABIIPT638T641	CST	9375	PRKCA/PRKCB	Rabbit	0.92176
GBL1153389	PKCBIIPS660	CST	9371	PRKCA/B/D/E/H/Q	Rabbit	0.97611
GBL1153357	PKCDELTAPS664	Millipore	07-875	PRKCD	Rabbit	0.96835
GBL1153396	PKCA	CST	2056	PRKCA	Rabbit	0.96973
GBL1153366	PKM2	CST	4053	PKM	Rabbit	0.97464
GBL1153634	PLCGAMMA1	CST	5690	PLCG1	Rabbit	0.95662
GBL1153635	PLCGAMMA1PS1248	CST	8713	PLCG1	Rabbit	0.95253
GBL1153611	PLCGAMMA2PY759	GeneTex	GTX133463	PLCG2	Rabbit	0.86366
GBL1153341	PLK1	CST	4513	PLK1	Rabbit	0.96221
GBL1153543	PMS2	Abcam	ab110638	PMS2	Rabbit	0.97023
GBL1153689	PORIN	Abcam	ab14734	VDAC1	Mouse	0.88164
GBL1153548	PR	abcam	ab206926	PGR	Rabbit	0.96453
GBL1153673	PRAS40	Life Technologies	AHO1031	AKT1S1	Mouse	0.96381
GBL1153340	PRAS40PT246	Life Technologies	44-1100G	AKT1S1	Rabbit	0.95977
GBL1153584	PRC1PT481	Aviva Systems Biology	OAAF05458	PRC1	Rabbit	0.94589
GBL1153407	PREX1	Abcam	ab102739	PREX1	Rabbit	0.95908
GBL1153327	PTEN	CST	9552	PTEN	Rabbit	0.95587
GBL1153531	PTPN12	Abcam	ab76942	PTPN12	Rabbit	0.96878
GBL1153419	PUMA	CST	4976	BBC3	Rabbit	0.90955
GBL1153412	PYGB	Sigma-Aldrich	SAB2900066	PYGB	Rabbit	0.92949
GBL1153784	PYGM	Novus	H00005837-M10	PYGM	Mouse	0.83345
GBL1153758	PYK2PY402	CST	3291	PTK2B	Rabbit	0.90417
GBL1153544	RAB11	CST	3539	RAB11A/B	Rabbit	0.95239
GBL1153393	RAB25	CST	4314	RAB25	Rabbit	0.97111
GBL1153512	RAD23A	CST	24555	RAD23A	Rabbit	0.96041
GBL1153441	RAD50	CST	3427	RAD50	Rabbit	0.90374
GBL1153657	RAD51	Millipore	ABE257	RAD51	Rabbit	0.95513
GBL1153386	RAPTOR	CST	2280	RPTOR	Rabbit	0.97338
GBL1153668	RB	CST	9309	RB1	Mouse	0.94907

GBL1153390	RBM15	Novus	21390002	RBM15	Rabbit	0.9745
GBL1153326	RBPS807S811	CST	9308	RB1	Rabbit	0.9515
GBL1153793	RHEB	R&D Systems	MAB3426	RHEB	Mouse	0.89554
GBL1153387	RICTOR	CST	2114	RICTOR	Rabbit	0.97344
GBL1153388	RICTORPT1135	CST	3806	RICTOR	Rabbit	0.97364
GBL1153765	RIP	CST	4926	RIPK1	Rabbit	0.95338
GBL1153766	RIP3	CST	13526	RIPK3	Rabbit	0.87759
GBL1153576	RPA32	CST	52448	RPA2	Rabbit	0.93282
GBL1153654	RPA32PS4S8	Bethyl	A300-245A	RPA2	Rabbit	0.94136
GBL1149522	RRM1	CST	3388	RRM1	Rabbit	0.96278
GBL1153514	RRM2	Life Technologies	PA5-27856	RRM2	Rabbit	0.97255
GBL1153342	RSK	CST	9347	RPS6KA1/2/3	Rabbit	0.97732
GBL1153603	RSK1	CST	8408	RPS6KA1	Rabbit	0.98124
GBL1153522	S100A4	CST	13018	S100A4	Rabbit	0.92894
GBL1153697	S6	CST	2317	RPS6	Mouse	0.96722
GBL1153328	S6PS235S236	CST	2211	RPS6	Rabbit	0.95176
GBL1153329	S6PS240S244	CST	2215	RPS6	Rabbit	0.94341
GBL1153713	SCD	Santa Cruz	sc-58420	SCD	Mouse	0.94016
GBL1153426	SDHA	CST	11998	SDHA	Rabbit	0.93479
GBL1153679	SF2	CST	14902	SRSF1	Mouse	0.96404
GBL1153637	SFRP1	CST	3534	SFRP1	Rabbit	0.95712
GBL1153790	SGK1	CST	12103	SGK1	Rabbit	0.86395
GBL1153788	SGK3	CST	8156	SGK3	Rabbit	0.90655
GBL1153631	SHCPY317	Bioss	bs-3413R	SHC1	Rabbit	0.9445
GBL1153403	SHP2PY542	CST	3751	PTPN11	Rabbit	0.94523
GBL1153542	SHP2	CST	3397	PTPN11	Rabbit	0.944
GBL1153422	SLC1A5	Sigma-Aldrich	HPA035240	SLC1A5	Rabbit	0.96179
GBL1153736	SLFN11	Santa Cruz	sc-136891	SLFN11	Goat	0.9408
GBL1153669	SMAC	CST	2954	DIABLO	Mouse	0.96776
GBL1153356	SMAD1	Epitomics/Abcam	1649-1/ab33902	SMAD1	Rabbit	0.96879
GBL1153344	SMAD3	Epitomics/Abcam	1735-1/ab40854	SMAD3	Rabbit	0.96589
GBL1153628	SMAD4	CST	46535	SMAD4	Rabbit	0.95658
GBL1153670	SNAIL	CST	3895	SNAI1	Mouse	0.95503
GBL1153696	SOD1	CST	4266	SOD1	Mouse	0.95294
GBL1153547	SOD2	CST	13194	SOD2	Rabbit	0.96029
GBL1153605	SOX17	Abcam	ab224637	SOX17	Rabbit	0.95316
GBL1153475	SOX2	CST	2748	SOX2	Rabbit	0.95913
GBL1153671	SRC	Millipore	05-184	SRC	Mouse	0.96219
GBL1153626	SRCPY416	CST	6943	SRC	Rabbit	0.95259
GBL1153330	SRCPY527	CST	2105	SRC	Rabbit	0.96534
GBL1153545	STAT1PY701	CST	9167	STAT1	Rabbit	0.91966
GBL1153405	STAT3	CST	4904	STAT3	Rabbit	0.96363
GBL1153553	STAT3PY705	Fisher	44-380G	STAT3	Rabbit	0.97153
GBL1153331	STAT5A	Epitomics/Abcam	1289-1/ab32043	STAT5A	Rabbit	0.95316
GBL1153336	STATHMIN1	Epitomics/Abcam	1972-1/ab52630	STMN1	Rabbit	0.94699
GBL1153526	STING	CST	13647	STING1	Rabbit	0.96684
GBL1153710	SYK	Santa Cruz	sc-1240	SYK	Mouse	0.93654
GBL1153672	TAU	Millipore	05-348	MAPT	Mouse	0.93802
GBL1153500	TAZ	CST	4883	WWTR1	Rabbit	0.89631
GBL1153425	TFAM	CST	7495	TFAM	Rabbit	0.97162
GBL1153391	TFRC	Novus	22500002	TFRC	Rabbit	0.95417
GBL1153773	TIGAR	Epitomics/Abcam	S1711/ab137573	TIGAR	Rabbit	0.95846
GBL1153708	TRANSGLUTAMINASE	Lab Vision	MS-224-P1	TGM2	Mouse	0.95215
GBL1153702	TRAP1	BD Biosciences	612344	TRAP1	Mouse	0.97451
GBL1153742	TRIM25	Abcam	ab167154	TRIM25	Rabbit	0.94835
GBL1153586	TRIP13	Invitrogen	PA5-52193	TRIP13	Rabbit	0.95017

GBL1153384	TSC1	CST	4906	TSC1	Rabbit	0.97026
GBL1153781	TTF1	Epitomics/Abcam	2044-1/ab76013	NKX2-1	Rabbit	0.92634
GBL1153332	TUBERIN	Epitomics/Abcam	1613-1/ab32554	TSC2	Rabbit	0.96676
GBL1153640	TUBERINPT1462	Abcam	ab109403	TSC2	Rabbit	0.96001
GBL1153577	TUFM	Invitrogen	PA5-27511	TUFM	Rabbit	0.95886
GBL1153724	TWIST	Santa Cruz	sc-81417	TWIST1	Mouse	0.92558
GBL1153597	TYRO3	Novus Biologicals	NBP1-28635	TYRO3	Rabbit	0.95669
GBL1153472	UHISTONEH2B	CST	5546	H2BC3	Rabbit	0.91562
GBL1153416	UBAC1	Sigma-Aldrich	HPA005651	UBAC1	Rabbit	0.82706
GBL1153732	UBQLN4	Santa Cruz	sc-136145	UBQLN4	Mouse	0.90654
GBL1153783	UGT1A	Santa Cruz	sc-271268	UGT1A1/3-5/7-10	Mouse	0.90739
GBL1153767	ULK1PS757	CST	6888	ULK1	Rabbit	0.95394
GBL1153785	UQCRC2	MitoSciences/Abcam	MS304/ab14745	UQCRC2	Mouse	0.84764
GBL1153487	UVRAG	CST	13115	UVRAG	Rabbit	0.95706
GBL1153333	VASP	CST	3112	VASP	Rabbit	0.97111
GBL1153404	VAV1	CST	2502	VAV1	Rabbit	0.91128
GBL1153334	VEGFR2	CST	2479	KDR	Rabbit	0.96263
GBL1153595	VEGFR2PY1175	CST	3770	KDR	Rabbit	0.89579
GBL1153581	VHL	CST	68547	VHL	Rabbit	0.9477
GBL1153705	VHLEPPK1	BD Biosciences	556347	EPPK1	Mouse	0.91646
GBL1153692	VINCULIN	Sigma-Aldrich	SAB4200080	VCL	Mouse	0.96148
GBL1153491	WEE1	CST	4936	WEE1	Rabbit	0.93775
GBL1153523	WEE1PS642	CST	4910	WEE1	Rabbit	0.95158
GBL1153769	WIPI1	CST	12124	WIPI1	Rabbit	0.94141
GBL1153838	WIPI2	CST	8567	WIPI2	Rabbit	0.9246
GBL1153735	XBP1	Santa Cruz	sc-32136	XBP1	Goat	0.93329
GBL1153335	XIAP	CST	2042	XIAP	Rabbit	0.95934
GBL1153726	XPA	Santa Cruz	sc-56813	XPA	Mouse	0.95004
GBL1153519	XPF	CST	13465	ERCC4	Rabbit	0.96223
GBL1153754	XRCC1	Invitrogen	PA5-29359	XRCC1	Rabbit	0.94529
GBL1153647	YAP	Santa Cruz	sc-15407	YAP1	Rabbit	0.9259
GBL1153343	YAPPS127	CST	4911	YAP1	Rabbit	0.96913
GBL1153348	YB1PS102	CST	2900	YBX1	Rabbit	0.97601
GBL1153638	YES1	CST	3201	YES1	Rabbit	0.95487
GBL1153499	ZAP70	CST	3165	ZAP70	Rabbit	0.92567
GBL1153593	ZEB1	CST	3396	ZEB1	Rabbit	0.94939
